# Multispectral Imaging for Skin Diseases Assessment—State of the Art and Perspectives

**DOI:** 10.3390/s23083888

**Published:** 2023-04-11

**Authors:** Mihaela-Andreea Ilișanu, Florica Moldoveanu, Alin Moldoveanu

**Affiliations:** Department of Computer Science and Engineering, University Politehnica of Bucharest, 060042 Bucharest, Romania; mihaela.ilisanu@upb.ro (M.-A.I.);

**Keywords:** multispectral imaging, dermatology, NIR imaging, skin cancer diagnosis, skin lesion assessment

## Abstract

Skin optical inspection is an imperative procedure for a suspicious dermal lesion since very early skin cancer detection can guarantee total recovery. Dermoscopy, confocal laser scanning microscopy, optical coherence tomography, multispectral imaging, multiphoton laser imaging, and 3D topography are the most outstanding optical techniques implemented for skin examination. The accuracy of dermatological diagnoses attained by each of those methods is still debatable, and only dermoscopy is frequently used by all dermatologists. Therefore, a comprehensive method for skin analysis has not yet been established. Multispectral imaging (MSI) is based on light–tissue interaction properties due to radiation wavelength variation. An MSI device collects the reflected radiation after illumination of the lesion with light of different wavelengths and provides a set of spectral images. The concentration maps of the main light-absorbing molecules in the skin, the chromophores, can be retrieved using the intensity values from those images, sometimes even for deeper-located tissues, due to interaction with near-infrared light. Recent studies have shown that portable and cost-efficient MSI systems can be used for extracting skin lesion characteristics useful for early melanoma diagnoses. This review aims to describe the efforts that have been made to develop MSI systems for skin lesions evaluation in the last decade. We examined the hardware characteristics of the produced devices and identified the typical structure of an MSI device for dermatology. The analyzed prototypes showed the possibility of improving the specificity of classification between the melanoma and benign nevi. Currently, however, they are rather adjuvants tools for skin lesion assessment, and efforts are needed towards a fully fledged diagnostic MSI device.

## 1. Introduction

Skin malformations are very common and, usually, they do not generate any discomfort to individuals affected, other than a possible aesthetic concern. In addition, malignant skin lesions are not easy to discriminate from benign ones with naked eye examination. This is one of the possible reasons that the screening of cutaneous lesions is frequently postponed and the correct diagnosis is found when the cancer has already spread to other parts of the human body. If it is detected in its incipient phase, skin cancer is treatable and survival rates are extremely high [1]. To ensure early skin cancer detection, screening should be accessible and very affordable for the general population [2]. Those needs require upgrading the current technology used in medical establishments. The development of better tools for dermatologists could improve the accuracy of cutaneous tissue analysis. Furthermore, the time assigned to the diagnosis process can be streamlined by using machine-based preliminary inspection and assistance.

There are numerous methods for skin evaluation and, in particular, the optical ones are preferred due to their non-invasive form. Dermoscopy, confocal laser scanning microscopy, optical coherence tomography, multispectral imaging, multiphoton laser imaging, and 3D topography are the most popular optical approaches for cutaneous tissue inspection [1,3,4,5,6]. Practical use is not common for many of these methods because of the novelty of the technique and, implicitly, the financial cost. In addition, the performance attained by each method varies, depending on the type of lesion analyzed. Therefore, a comprehensive method for skin analysis has not yet been established.

The gold standard in skin tissue assessment, histological examination, can favorize the spreading of malignant cells because of the high metastatic activity of melanoma. Furthermore, it involves the complete excision of the lesion which generates a certain discomfort and a financial cost, which can be substantially increased if the patient has multiple dysplastic nevi [7]. Accessibility of skin tissue facilitates the implementation of colorimetric and texture analyses [8]. The use of an optical visualization technique could facilitate dermal evaluation and sustain a more accurate diagnosis with minimal distress. Furthermore, optical methods are more time-efficient since the diagnosis can be assessed in minutes versus days in the case of histological inspection [8].

Multispectral imaging (MSI) is a technique used to collect and analyze images from several spectral bands or wavelengths of light. The sensors used to capture a scene or an object of interest have a high sensitivity for radiation with wavelengths even beyond the visible range spectrum (400–700 nm) for the human eye [9,10]. The spectral channels involved may range from 400 to 2500 nm, from the visible light spectrum to infrared. The method allows the analysis of additional information about the object or scene being imaged, such as its physical, chemical, or biological properties. Multispectral imaging is commonly used in a wide range of applications, including agriculture, environmental monitoring medical imaging, and remote sensing [11]. In medical imaging, multispectral imaging can provide valuable information about the structure and function of tissues and organs, helping clinicians to make more accurate diagnoses and treatment decisions [9]. The number of spectral bands of MSI systems is limited to a maximum of ten, and when dozens or even hundreds of narrow spectral channels are used the approach is called hyperspectral imaging (HSI) [8]. Both types of technique were implemented in dermatology, but since the HSI prototypes involve a large quantity of data to process, which can lead to redundancy among the information collected [12], the present review will be focused on the MSI architectures. The multispectral imaging approach for dermatology involves sequential illumination of a skin region of interest with light of several wavelengths, measuring reflected radiation and thus evaluating the illuminated tissues [13]. Systems built using this technique have shown good potential in early skin cancer detection, as well as promising cost efficiency. Compared to other techniques, MSI systems have a simple operation principle and can supply information regarding chromophores’ concentrations, even for deeper tissues, to sustain a proper diagnosis.

The functioning of MSI devices for dermatology is based on light–tissue interaction properties. The skin has a heterogenous structure and its behavior when penetrated by radiation is strongly related to the molecular content. The way in which chromophores such as oxy and deoxyhemoglobin, melanin, water, beta-carotene, bilirubin, collagen, or lipids absorb and scatter light is reflected in the appearance of the skin [14]. Hence, their concentrations are relevant to certain pathologies of skin. The wavelength of light determines the depth of penetration into the tissues. A depth of 2.5 cm can be attained by radiation with a wavelength of 850–900 nm [15]. Subcutaneous information is important since it can reveal details that are inaccessible with the naked eye or with the standard dermoscopic approach. The output of an MSI system is the spectral cube, which contains spatial and spectral information about the sample analyzed [3]. Spectral images of a lesion can be used to distinguish between healthy and unwholesome tissue. The set of parameters that can be extracted is called the spectral signature of the analyzed sample. These data can be easily used as input for algorithms that perform automated classification.

This review aims to synthetize and describe the efforts made to develop multispectral imaging systems for skin lesion evaluation in the last decade. The focus is on the hardware characteristics of the produced devices, their advantages and limitations, and particularly their performance for dermatology.

In Section 2, we present the various current imaging techniques for skin tissue analysis. We present their operation principles, advantages, and limitations, as well as how frequently they are used in clinical practice. Thus, MSI systems (which are the focus of this paper and will be detailed in the next sections) will be better understood in this context.

In Section 3, we describe the commercially available MSI systems for dermatological applications and the prototypes built to overcome the disadvantages of the first mentioned devices.

In Section 4, we discuss the current hardware features and the attained performances of both commercial and prototype MSI systems. 

## 2. Approaches of Skin Lesion Analysis

Skin is the largest organ of the human body and its screening for medical purposes first involves a visual inspection. Naked eye investigation is not enough to provide an accurate diagnosis for a skin malformation, because the procedure is extremely sensitive to the expertise and experience of the physician who performs the task. Especially in skin cancer detection, the accuracy of diagnosis is extremely important because the mortality of this disease can be significantly decreased if it is recognized at an early stage. In addition, malignant lesions often have common characteristics of other benign skin malformations, and subcutaneous or cellular-size features can be a better decision criterion.

Due to cutaneous easy-access properties, many imaging techniques were established to improve the accuracy of dermatological examinations [16]. The phenomenon of interaction with light, from visible to extended near-infrared spectra, was used to develop optical tools for clearing the tissues. Each tool aimed to generate more information about the structure and distribution of the main cellular components of the skin. 

Dermoscopy offers a clear visualization of superficial dermis, due to magnification and polarized light. Deeper layers of the skin can be inspected with high resolution using optical coherence topography or confocal laser scanner microscopy. Multiphoton topography can provide 3D visualization at cellular level, while chromophore mapping can be performed using multispectral imaging. The height of the skin surface can be retrieved using the 3D topographic technique of fringe projection. The functional principles and the main advantages and limitations of those skin analysis approaches will be further described. Their hardware characteristics and performances reported for melanoma detection are listed in Table 1; also, Table 2 summarizes the main advantages and limitations, as well as future possible improvements, of the imaging techniques used for dermatology purposes. 

### 2.1. Dermoscopy

Dermoscopy, or epiluminescence microscopy with incident light, is a non-invasive technique to analyze skin lesions. The incident light can be either polarized or non-polarized, and, in this case, an interface fluid is used to reduce the specular reflectance [4].

Distinguishing between malignant and benign lesions is the aim of the investigation of pigmented skin lesion [21]. Dermoscopy is a standard technique in the assessment of skin diseases and is associated with increased accuracy of diagnosis both for pigmented and non-pigmented lesions [4]. Melanoma detection is 50% more precise with dermoscopy than solely visual inspection of skin tissue by a dermatologist. Thus, medical professionals are trained to use specific decision algorithms, such as the ABCDE method, the Menzies method or the seven points list of Argenziano [1]. Pattern analysis of a lesion was established in 2003 to be the most sensible and specific modality to evaluate the malignity of a suspicious spot on dermal tissue [23]. Thus, investigation of the structure of several types of skin disease was a permanent concern in the dermatology field. Apart from melanoma characteristics, dermoscopic properties of the most frequent subtypes of skin cancer, such as basal cell carcinoma and keratinocyte tumors, were identified [4]. 

The dermoscope is the device used by dermatologists to observe a skin lesion. Most often, the device has a source of polarized light and a system of lenses which offers 10× magnification. Digital dermoscopes have a camera incorporated, and images of the skin lesions can be acquired to follow their evolution or to detect the occurrence of new nevi. These devices are quite easy to use and do not increase the time of screening. The whole body can be analyzed in 30 min. Thus, their main advantages are related to the increased rate of accuracy compared to the naked-eye inspection performed by a specialist, as well as the efficient use of time.

Dermoscopy can be optimized to refine the diagnosis by improving the decision algorithms. Its disadvantages are related to limited visualization in depth of the skin. Only superficial layers could be analyzed: epidermis, epidermis–dermis junction, and superficial dermis. Dermoscopy does not provide transversal information. The technique is a contact method of evaluation because it is necessary for the device to touch the skin of a patient if using polarized light, or for the skin to be immersed with an interface fluid (oil or water) when using non-polarized light. Screening of the whole body is possible and easy to implement with a dermoscope, but it generates substantial data to interpret. This technique does not significantly reduce the number of biopsies performed on benign nevi [3]. 

### 2.2. Confocal Laser Scanning Microscopy

Confocal laser scanning microscopy (CLSM) is a non-invasive technique used for skin tumor examination at cellular level, reaching a resolution of 0.5–1 µm [4]. A conventional system includes a laser diode of near-infrared light with wavelengths around 830 nm as an illumination source, a microscopic architecture of lenses which assures that the light reflected from the analyzed tissue is focused through a punctiform aperture, and a detector, commonly a CCD camera [3,6]. The name confocal is a consequence of the fact that the planes of the light source, the punctiform opening and the region of interest at the level of the skin are conjugated [21]. In the resulting grayscale images, the bright white regions correspond to melanin, keratin, dermis collagen, and inflammatory cells, which have higher refractive indexes [5]. Thus, the structure of epidermis and the morphology of the cells can be observed with clarity [3]. The confocal images are captures of horizontal planes from different sections of the skin, parallel with its surface [5]. The process of obtaining these images is termed optical sectioning and the thickness of the obtained vertical stack is 2–5 µm [6]. Recording these stacks over time enables the acquisition of 3D information about a skin lesion [3,17].

There are two approaches of confocal microscopy: reflectance and fluorescence CLSM, both applied in dermatology [3]. Reflectance confocal microscopy (RCM) is more suitable for in vivo skin visualization than the fluorescence modality which involves using specific dyes in the sample of tissue [3]. There are a variety of staining techniques that enable access to information about skin structure to identify a single molecule at cellular level [3]. The fluorescence approach has the capability to offer 3D investigations, so it is widely used for ex vivo examination or in operating rooms, when certain fluorophores are inserted into the tissue for tumor demarcation [3].

The quality of images obtained by CLSM is compared with the histopathological results, in terms of features highlighted as suspicious lesions [6]. Thus, as a supplement to dermoscopic inspection, CLSM can improve the accuracy of detection of melanoma lesions without malignant features [4,17]. Further, it has been shown that the margins of tumors can be precisely detected [1], and that the number of biopsies of benign nevi can be reduced using confocal microscopy [4]. Dynamic physiological and pathological changes in a skin lesion can also be observed and the treatment can be adapted concurrently with the time evolution of the damaged tissue [3,4]. 

This method is only suitable for skin tumors which need a reduced depth focus [4]. The maximum depth penetration of tissue is limited to only 200–300 µm [1,4,5]. Clinical use of this method is not frequent because of the financial and time cost needed for implementation and training of dermatologists [1,16,17]. Commercially available RCM devices are VivoScope 1500 and VivoScope 3000 (Caliber Imaging &Diagnostics, Inc., New York, NY, USA) [5,17]. The latter is a handheld device that facilitates the assessment of skin lesions using RCM. It is necessary that the device is adhesive to the skin; thus, this is a contact technique. False-positive results can be easily achieved in the case of curved surface of the skin, nevi with a high degree of dysplasia, or when the diagnosis feature is below the papillary dermis [1,17]. Furthermore, it is a very time-consuming procedure in terms of image processing, 7–10 min [1]. However, the high-quality images obtained represent a main advantage of the CLSM, which can compensate the drawbacks [1]. 

### 2.3. Optical Coherence Tomography

Optical coherence tomography (OCT) is an imaging technique first established in 80′ and it is widely used in ophthalmology and, only recently, in dermatology [18]. An OCT system relies on Michelson interferometry and uses an NIR laser source to illuminate the sample and obtain 2D or 3D cross-sectional images from the reflected signal [4,17,18]. Using a beam-splitter, two light aims are created: a reference beam and an object beam. The object beam focuses on the sample and is then backscattered. The reflected beam recombines with the reference one on the photodetector, where an interference image is constructed if path lengths of both beams match the coherence length of light [17,18]. Differences between refractive indexes of skin components generate contrast in the resulting grayscale images [5,19]. Three-dimensional visualization of analyzed tissue is possible through OCT by acquiring sequentially horizontal or en face images at different depths [3,24]. The penetration depth through the skin tissue was demonstrated to be related to the wavelength of radiation used by the laser source [5]. Many OCT systems use wavelengths from the upper part of the NIR region, with a value of 1300 nm being the most frequently chosen. Thus, they reach a penetration depth of 1–2 mm, which is considered an intermediate level of skin lesion assessment [3,18,19]. Deeper skin structures, such as lower reticular dermis and even movement of blood vessels, can be visualized thanks to OCT devices, but axial and lateral resolutions are limited to 7.5 µm and 5 µm, respectively (VivoSight, Michelson Diagnostics Ltd., Orpington, Kent, UK) [3,5,18]. Greater resolutions, of 3 µm for both axial and lateral directions, come with the drawbacks of only 570 µm penetration depth (Skintell, Agfa Healthcare, Mortsel, Belgium) and a significantly reduced field of view, of only 1.8 mm × 1.5 mm versus 6 mm^2^ for the VivoSight device [18].

Although the penetration depth is better than that achieved through RCM, OCT only allows the investigation of a skin lesion at structural resolution [4]. This means that it cannot be used for examinations at cellular level, and that the resolution is lower than that of histology or even RCM [1,4]. High-definition OCT (HD-OCT) based on frequency-domain OCT (FD-OCT), dynamic OCT (D-OCT) based on speckle-variance OCT (SV-OCT) [3], and the very recently developed line-field confocal OCT (LC-OCT) [24] are the variants of OCT systems architectures conducted to improve this limitation. HD-OCT demonstrated a higher sensitivity than CLSM or multispectral imaging systems and a strong correlation between captured images and histological features [18]. SV-OCT is well known for enabling microvasculature imaging. Tumor growth occurs with the extension of blood vessel supply; thus, D-OCT improves earlier detection of skin cancer [1,17,18]. LC-OCT is an approach in the time domain. Vertical section images are acquired using a line source for sample illumination and a line camera for detection. Although the penetration depth is poor and field of view 3D is 1.2 × 0.5 × 0.5 mm^3^, the morphological details captured in 3D format show that this emerging technique has great potential for dermatological applications [24].

OCT can be a good tool for skin tumor real-time evaluation and during a treatment session, without altering the tissue [25]. Deep margins of a skin tumor can be detected [6] and the number of unnecessary biopsies can be reduced using OCT [1]. However, it has some disadvantages, since OCT methods registered lower specificity for early melanoma detection, meaning a high false-negative rate for thin melanomas [17,18,19]. In addition, a high false-positive rate for dysplastic nevi was reported, which can lead to mistaken diagnosis because the thickness of the tumor and the configurations of its margins are important decision factors [17]. Clinical use of the technique is not very common as a consequence of the high financial and time cost of the implementation, and the required special training for dermatologists [4,6,16].

### 2.4. Multispectral Imaging

Multispectral imaging (MSI) systems allow the simultaneous collection of spectral and spatial information from human tissues [6]. They measure the signal reflected or transmitted by the analyzed sample after exposure to radiation from certain spectral bands [3]. For dermatology applications, the wavelengths of the measured radiation correspond to peaks of absorbance or/and reflectance of the principal chromophores of the skin. A chromophore is a molecule from skin that varies in concentration in different pathologies [3]. The principal chromophores are oxy and deoxyhemoglobin, melanin, water, collagen, and bilirubin [3]. 

There are two MSI approaches for skin lesion. Active systems use mostly LEDs, in different configurations, as a source of incident light on the skin lesions. At the sensor of a detector, the radiation (having several different wavelengths) is then collected, reflected by the region of interest of the cutaneous tissue, and sequentially used for its illumination [3]. Passive systems have a broadband source of light and the reflected light is sequentially filtered before reaching the detector. The filtering is performed by mechanically rotating optical filters with spectral narrow bands, also called filter wheels, or by electronic tunable filters: liquid crystals or acusto-optical tunable filters (LCTF and AOTF, respectively) [26].

The output of an MSI is “the spectral cube”, which consists of a sequence of images corresponding to the wavelengths of light used for illumination. Thus, the value of a pixel represents the intensity of light reflected by the sample analyzed at a specific wavelength of the incident light, in a spectrum range from visible to near-infrared radiation (400–950 nm) [19]. 

The MSI method was used for the assessment of pigmented lesions of skin for early detection of melanoma [27]. As commercially available systems enable the collection of information from 2.5 cm below the skin surface [1], benign and malignant nevi can be distinguished due to their spectral characteristics. Moreover, the vascular depth of the skin lesion can be estimated and, thus, can contribute to dysplastic nevi classification, as well as evaluation of water content, which is known to be correlated with angiogenesis and so with tumor growth [3,6,27]. In this type of system, spectral images are automatically processed in order to obtain statistical pattern recognition in a skin lesion and to observe morphological features related to a possible skin disorder [1].

Low specificity, or the high percent of non-malignant lesions classified as melanoma, is the main limitation of the available MSI devices and the reason that they are not implemented in clinical use [8]. In addition, the 3D visualization of skin tissue is not very well investigated because of the reduced contrast of images captured using radiation from upper NIR spectrum. Multispectral optoacoustic tomography (MSOT) is a technique recently used in dermatology, which can provide 3D visualization. Using pulsed NIR to generate acoustic waves, under-skin details from a maximum of 1 cm depth are depicted, and the resolution reached is only 60 µm [5,28].

Hyperspectral approaches for skin inspection have the same operation principle as MSI systems, but the main difference is related to the much higher number of narrow spectral bands involved in the acquisition process. The absorption information about chromophores can be extracted by a snapshot hyperspectral camera with only one exposure, minimizing the risk of motion artifacts in images [29]. Besides skin cancer detection, the possibility of monitoring skin tissue activities, such as a response to a vascular occlusion, wound examination, and heartrate assessment, was also investigated and provided good preliminary results [29]. 

Ding et al. [30] chose a different approach for implementing an MSI device. They sought to overcome the limitations of traditional MSI configurations in terms of size and time consumption, and they built an optical MSI chip that mimics the compound eye of an insect. A linear variable filter and lens were used to assemble the MSI chip and to replace the use of multiple filters or different wavelengths of light for illumination of the target region of skin. The chip was placed between two sets of lenses in front of a smartphone camera, while the orthogonal polarization approach was implemented for the ring-shaped illumination source, placed in front of the first lens. As each eye of the MSI optical chip responds to a specific spectral band, nine different images (for wavelengths between 400 and 700 nm) were acquired to form the spectral cube [30]. Image processing was performed for the data collected for a pigmented nevus of 10 mm diameter. Distribution maps of melanin and hemoglobin concentrations were retrieved, and Fisher linear discrimination was completed to solve the pattern recognition problem [30]. The optical resolution of this new MSI system is limited to 100 µm. The preliminary results showed good potential in pathological versus healthy tissue examination and proved the possibility of implementing a snapshot MSI which can record the spectral cube in a single exposure [30].

MSI is a recently developed technique for dermatology purposes, so its improvement is an important concern due to the proven potential in melanoma detection until the present. Multiple prototypes of MSI systems were created, but the optimum configuration of a device which would offer the possibility of visualizing deeper layers of skin lesions and evaluating the malignancy level with great sensitivity and specificity is still under investigation.

### 2.5. Multiphoton Laser Imaging

Multiphoton laser microscopy or multiphoton topography (MPT), as it is more often called in the literature, is an imaging technique applied for skin tissue and reaches a resolution of cellular level [17]. It is based on the interaction between photons and fluorophores from skin, such as melanin, keratin, collagen, and elastin [5,6]. Fluorescence occurs due to excitation of two or more photons in the near-infrared region using a femtosecond laser as a light source [6,17]. Thus, a maximum of 200 µm depth through skin tissue can be reached, so the epidermis and upper dermis can be visualized, performed using optical sectioning without a confocal pinhole [17]. 

In the high-resolution images of skin diseases, melanoma features were observed, such as increased number of melanocytes, large intracellular distance, cell pleomorphism, and structural disorder [17]. The morphologic characteristics captured through MPT were considered comparable to those achieved in histological examination [6].

A commercial system for skin lesion inspection was developed, called DermaInspect (JenLab, Berlin, Germany), which employs MPT combined with OCT. The device has a field of view of 350 × 350 × 200 µm^3^ and reveals subcellular details of skin lesions [5]. For the classification problem of malignant and benign nevi, it was reported that a sensitivity of 71–95% and a specificity of 69–97% were attained [17].

The main disadvantage of this method is its high cost of implementation as well as limited depth penetration, and the fact that resolution can be easily affected by motion artifacts [17]. However, accuracy for skin cancer detection was found to be similar to that of CLSM, and even the 3D representations and tumor depth estimation were recognized to be more accurate with MPT [6].

### 2.6. 3D Topography—Fringe Projection

Skin relief changes when a malformation appears in the skin tissue. Also termed topography, this feature of skin cannot be straightforwardly analyzed, but it can be very useful in the assessment of severity of a disease [3]. The skin surface height can be retrieved in-vivo using several 3D topographical imaging techniques, but fringe projection is the most popular and efficient method [3]. To extract significant information about a skin disease, the triangulation measuring principle is used. A whole fringe pattern is projected on the region of interest and a defined configuration of cameras captures the reflected light. Only this technique meets the ISO standards for roughness evaluation and commercial systems for skin inspection are available [3].

Skin cancer detection using machine learning was also implemented. A handheld prototype was developed, and 3D reconstructions of multiple skin lesions were acquired with a spatial resolution of 15 µm in less than 5 s per lesion [22,31]. Parameters such as area, perimeter, and volume were found to be significantly distinct in the case of benign versus malignant lesions, and the supervised classification reported an increased specificity than other similar studies did [22]. The main disadvantage of the method is its sensitivity to motion of the patient during the imaging procedure, which can determine artifacts in the final images.

## 3. Multispectral Imaging Systems

The benefits of multispectral imaging for dermatology have been thoroughly explored in the last decade. Sensitivity and specificity were reported to be higher when a multispectral imaging system was used for making the decision to perform biopsy for a suspicious lesion than in the case of analyzing only dermoscopic images [32]. Although commercial systems were produced, they have been helpful in the assessment of skin malformations only as screening devices. Lower specificity was reported for some of them, and high prices or bulky architecture were the factors that prevented their intense clinical use.

In this section, we present some of the outstanding developed MSI systems as devices for dermatological purposes. The analysis is conducted in two parts: the first depicts the characteristics of commercially available systems, and the second is focused on the emerging prototypes aimed at offsetting the drawbacks of current technologies. 

Studies included in this review were selected from multiple databases, such as PubMed, Web of Sciences, IEEE Xplore, Science Direct, Springer, SPIE, and Wiley. The covered studies were collected from the search using keyword filters, such as multispectral imaging, dermatology, skin cancer detection, and skin lesion assessment. The period between 2012 and 2022 was chosen to survey the evolution of multispectral imaging systems created for dermatological purposes.

**Figure 1:**
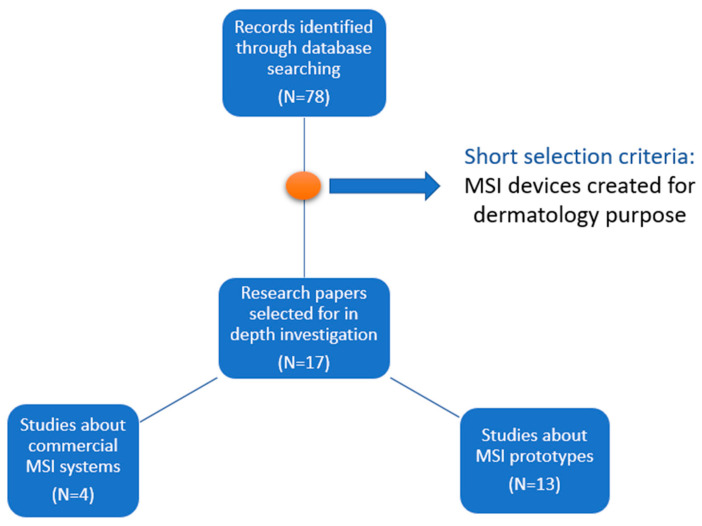
Flow diagram of the study selection process included in this review.

From the 78 research papers related to such topics, we selected and investigated 17 papers in depth: 4 describing commercially available systems and 13 prototypes of multispectral imaging systems built for skin analysis. In the short selection, only the papers that describe multispectral imaging systems created for dermatology purposes were included. Figure 1 summarizes the selection process of the studies included in this review. For each system, we analyzed its hardware components, its performance in discrimination between malignant and benign nevi, and the advantages and limitations resulting from its clinical use. Based on the individual analysis of the selected systems, we created a comparative analysis and synthesized the state and perspectives of the field.

### 3.1. The Advantages of Using Multispectral Imaging Systems for Dermatology

Multispectral imaging systems are a non-invasive, non-ionizing and non-contact approach for skin inspection. Even if these characteristics are not unique among other techniques applied in dermatology, they are very important features that an ideal system for skin inspection must satisfy. The radiation used for illumination in MSI devices does not alter the tissue in any way and it is safe to be used for anyone from the general population.

The main advantage of an MSI system is the penetration depth of the skin tissue, which can reach 2.5 cm below the skin surface. This is possible because light with wavelengths from the near-infrared spectrum is involved in the process of data acquisition. In addition, qualitative information can be retrieved about a skin lesion, such as the approximate depth of the lesion, due to sequential scanning for different spectral bands. As the wavelengths of light used for illumination are increased, the sub-surface details are revealed. The spectra collected from the visible region of spectrum are also crucial for the segmentation step, when the demarcation edges are established between healthy and pathological skin tissue.

Compared to other methods developed for dermatology, the MSI approach has a fast acquisition procedure, less than 10 s per lesion. This fact makes it convenient for screening patients with multiple nevi. The image processing is also fast and can be easily combined with automated classification to obtain a primary diagnosis for a skin lesion. Chromophore mapping is one of the main tasks performed using an MSI system and facilitates choosing a proper diagnosis by the dermatologist. Structural and morphological details, as well as molecular concentrations, can be evaluated using MSI devices.

### 3.2. Commercial Systems

MelaFind (MELA Sciences, Inc., Irvington, NY, USA) is one of the first commercially available devices for skin cancer diagnosis. Ten multispectral images are used as input for linear classification algorithms, resulting in a binary output for each skin lesion analyzed. The suspicious area of tissue is binary categorized regarding the level of malignity that can be low or high. The result given by this equipment is based on a statistical analysis to determine the morphologic disorganization of the lesion; for this reason, it is referred as a “blackbox” technique [1,33]. Even if the use of this device has been associated with a reduced number of false-negative results, the significant number of false-positive outcomes led to unsustainable clinical use [6]. It was concluded that low specificity (the ratio between the number of lesions classified as non-melanomas and the real number of non-melanoma lesions) of MelaFind could increase the screening cost and the procedures performed in order to find the precise diagnosis for a malformation of skin [34]. However, it was also highlighted that the very good sensitivity in detecting early melanomas may compensate the lack of specificity, especially in the case of a patient with numerous dysplastic nevi [1]. A comparative study of MelaFind, as a representative of MSI, and VivaScope 1500 for reflectance confocal microscopy, as approaches in dermatology, highlighted the superiority of the latter in terms of sensitivity and specificity [35]. For this reason, MelaFind was only considered as a clinical adjuvant device, and not a screening device [35].

SIA, from the name of the device SIAscope (Astron Clinica, Cambrige, UK, 2002) that stands for spectrophotometric intracutaneous analysis, is a method of assessing skin lesions using a handheld device [1]. Mapping the distribution and position of main chromophores of the skin is computed due to the eight spectral images acquired after sequential illumination with light from visible to NIR range. A melanoma risk score is constructed and a binary output is generated for each lesion, meaning high or low risk of melanoma. The statistical algorithm is named MoleMate, as is the recent version of the equipment. As with MelaFind, it is an expensive device and clinical studies showed very low specificity. Thus, several research papers suggested that the use of this equipment is not justified, because it did not significantly improve the diagnosis accuracy compared with dermoscopy [1].

DermoSight (Balter Medical AS, Bergen, Norway, 2017) was used in the study [36] of Stamnes et al. to discriminate between benign and melanoma tissue with high sensitivity and specificity, on both a small and a larger data set of 712 skin lesions. The classification was performed using a clustering method. Eighty-six parameters, related to physical and morphological properties of skin tissue optical characteristics, were computed from the 30 images acquired for each lesion. The blood content, melanin optical depth, and estimation of thickness for different layers of skin were ensured as parameter maps due to a complex and precise hardware architecture. In the source of illumination, the LEDs were set at certain angles from the skin surface to provide a greater depth penetration, and five mirrors ensured the capturing of the same skin area from three different directions. Their approach demonstrated great potential in improving cancer detection in the dermatology field, but validation of the technique performed on even larger datasets is needed. Even if its skin cancer automated evaluation algorithm is not published, the equipment was used for more than 120,000 commercial assessments of skin lesions [37].

Demetra (Barco, Kortrijk, Belgium, 2019) is a multispectral dermoscope launched very recently on the market. More than a handheld device for image capturing, the equipment of Demetra includes a Cloud-platform where the patients’ data are stored and can be easily accessed any time by physicians. Through the touchscreen display, dermatologist can rapidly measure and generate three chromophore maps to categorize the analyzed skin malformation. Thus, the patient receives fast feedback that can be sent to them in a digital format or be further analyzed by their physician. Melanin pigment, blood supply, and scattering contrast of the lesion can be individually assessed due to illumination with several spectral bands, and the final diagnosis can be more precise. Therefore, this type of visualization of subcutaneous structures from up to 2 mm depth was demonstrated to enable an analysis of basal cell carcinoma and Bowen’s disease lesions [31]. Knowing the light absorption properties of skin absorption, and the fact that the wavelength of radiation determines the penetration depth of the tissue, spectral signatures for each type of skin malformation were computed. The Beer-Lambert law is used for modeling the interaction of light and skin tissue, the attenuation being proportional to the concentration of the three main absorbers from the skin. Thus, their concentrations are retrieved on the assumption that the optical path value is constant for the entire field of view, and that absorption is the only factor that affects light behavior. The resulting maps enhanced the vascular patterns specific to assessed skin pathologies and made their recognition easier and clearer [31]. Images provided by the Demetra device were used in a recent study as a dataset to evaluate the performances of two deep neural networks on classification problems between multimodal images of different skin lesions [38].

**Figure 2:**
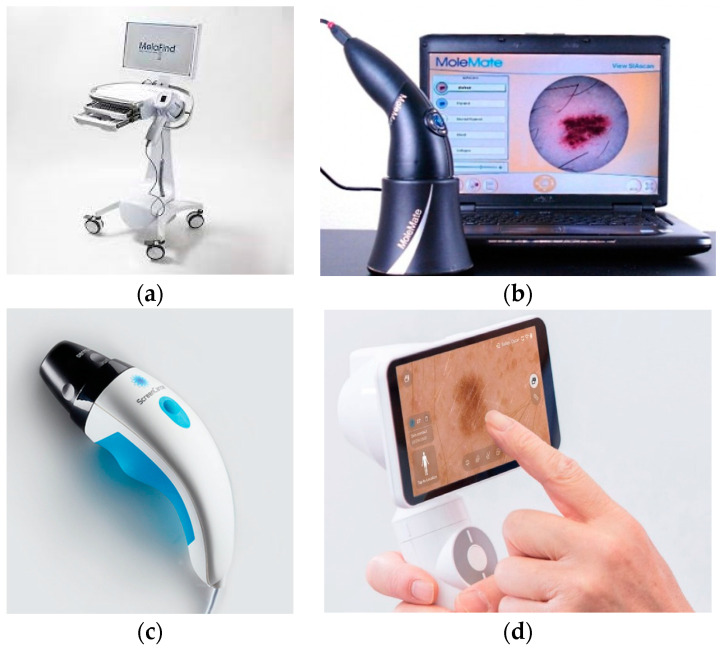
Multispectral commercial systems available for dermatology: (**a**) MelaFind [39], (**b**) SiaScope/MoleMate [40], (**c**) DermoSight [41], (**d**) Demetra [42].

Images of the multispectral commercial systems available for dermatology are presented in Figure 2. Table 3 summarizes the main advantages and limitations of the commercial MSI devices created for dermatology purposes. Features regarding hardware components, performance, and costs inherent to all systems described above are listed in Table 4.

### 3.3. Prototypes 

To offset the various disadvantages of the current MSI products for skin lesion inspection, different prototypes were developed, and their performances measured in some clinical studies exceed the current level of efficiency.

In this section, 13 MSI systems are presented in terms of hardware features and capabilities attained in discrimination between healthy and diseased skin tissue. 

The melanoma lesions, despite a benign nevi lesion, tend to still be visible in multispectral images acquired in the upper range of NIR spectrum. Using an MSI system it was proved by Pelagotti et al. [46] that the structure of malignant tissue can be still distinguished in the image corresponding to 950 nm, while the benign melanocytic nevus is completely vanished after 850 nm. It was noted that, in this way, depth information about skin tissue can be accessed, which is inaccessible through conventional dermoscopy. 

The differentiation parameter p between melanoma and non-malignant nevi, introduced by Diebele et al. [47], had been established using the spectral images acquired at only three wavelengths from the 51 images that were collected in the spectral range 450–950 nm. It was computed using the following formula:

$$p=OD_{650}+OD_{950}-OD_{540},$$

where $OD_{\lambda }=-\log\left[I\left(\lambda \right)-I_{0}\left(\lambda \right)\right]\;$ stands for optical density images, and $I\left(\lambda \right),\;I_{0}\left(\lambda \right)$ are the intensities of the light reflected from the skin and a white reference, respectively, for wavelength $\lambda \;\in \;\left\{540\;\mathrm{nm},650\;\mathrm{nm},\;950\;\mathrm{nm}\right\}.$ The choice was in accordance with the variation in absorption of the main chromophores of skin in the VIS-NIR (visible-near-infrared) spectrum. For hemoglobin, maximum and minimum absorbance wavelengths of 540 nm and 650 nm, respectively, were selected. The image corresponding to 950 nm was selected to express the alterations of chromophore concentration of deeper skin tissue. It was shown that higher values of the parameter were associated with melanoma lesions, and the values registered for nevi were comparable with those obtained for the surrounding skin of the lesion. Although the validation data set of the developed method was small, the results were particularly good and through the entire last decade, inspired many researchers to improve MSI solutions for dermatology applications.

Improving the specificity of MSI approaches in dermatology is required in order to increase the number of this type of device in clinical use. The information that can be accessed through sequential illumination and collecting the reflected spectra is superior to that gained with conventional dermoscopy. Pigmented structures can be clearly visualized while mapping a certain chromophore content, such as melanin, and their morphology can be further correlated with a corresponding skin pathology. An imaging system with this aim was described by Kapsokalyvas et al. in their study [48]. Five images are acquired for different states of polarizer and analyzer position and illumination settings. Melanin, blood, and scattering contrast maps are provided using several algorithms. The structures with high melanin content, the blood supply of the lesion, and the texture of the corresponding cutaneous area can be easily identified in those images. The device design makes it easy to attach to a dermoscope, demonstrating the possibility of increasing the accuracy of dermatological diagnosis with low-cost equipment [48].

Delpueyo et al. proposed and validated an MSI solution for classification between malignant and benign skin malformations with greater performances than the commercially available devices [49]. Their prototype allowed the acquisition of eight spectral images, corresponding to each of the wavelengths used for illumination, and computed two sets of images related to absorbance and reflectance of the light. To determine the difference between healthy and pathological tissue, 15 parameters were extracted as statistical descriptors from three categories of parameters: the pixel-by-pixel spline interpolation values, color features in CIELAB coordinates of the lesions, and the empirical parameters related to reflectance. The algorithm used for classification consisted of sorting the parameters decrescent to the number of malignant lesions that could be discriminant by it. Then, sensitivity and specificity were calculated for different combinations of these parameters, starting with the combination of the two that generated the greatest performance alone in separation of benign and malignant lesions. It was concluded that using textural parameters had improved the detection of melanoma and basal cell carcinoma. This system, that uses the radiation from VIS to NIR spectra, was further used in combination with another MSI device operating in the exNIR range and was presented in Rey-Barruso et al. [20]. Their approach aimed to expand the information about a lesion by accessing deeper layers of skin due to increased wavelengths of light involved in illumination. The previous device played an important role in the segmentation process, since the margins of a skin tumor are faded in images corresponding to higher spectral bands. Absorbance and reflectance images were computed to enable the classification task between malignant and benign tissue. The statistical descriptors mean, maximum, minimum, and standard deviation, as well as energy, entropy, and skewness, were calculated based on the corresponding histograms for each spectral image of a lesion. A principal component analysis (PCA) determined the accuracy of threshold values chosen for descriptors to be corelated with malignant or non-malignant cases. The classification was performed using a support vector machine (SVM) algorithm, using the seven descriptors found to be most relevant in accomplishing a greater specificity than the one registered in previous studies [20].

One of the first approaches to development of an MSI smartphone-based system was the device created by Kim et al. and described in [50]. They built an MSI module that can be attached to a smartphone and collect spectral images of a region of interest (ROI) of the skin. An Android application controlled the capturing and data transfer to the platform used for image processing. The MSI module (a collection of filters placed in a circular-shaped mount that can be rotated) enabled sequential illumination with radiation possessing a wavelength from 440 to 690 nm. A cube of nine images was obtained and spectral classification was applied. The spectral angles measure was used to achieve the spectral signatures for both nevus and acne regions. The achievement of spectral signature at every pixel in a ROI made a detailed analysis of the skin lesions possible. The severity of the acne was accurately quantified, and the nevus regions were precisely depicted from the normal skin. Thus, the potential of MSI for the assessment of skin diseases was again demonstrated.

The excellent capabilities of smartphones to capture images and their availability nowadays has raised interest among researchers in using them as tools for dermatological screening. Although numerous mobile applications were developed and there are even commercially available options, their performances are still debatable [51]. The task of discriminating between a malignant malformation of skin and a benign one was improved by evaluating the values of concentration of the chromophores in each point of the region of interest (operation called frequently mapping) of the cutaneous tissue [33]. Another approach, based on spectral properties of skin, similar to the MSI technique, is the autofluorescence photobleaching [52]. It was observed that, after illumination with radiation of short wavelengths, around 400 nm, a percentage of it is backscattered as radiation with longer wavelength, and this phenomenon is called autofluorescence. The emission is not completely stopped as the excitation is interrupted, so “fingerprints” in the measurements of the light re-emitted by the tissue can be seen. The photobleaching “fingerprints” were used to discriminate between healthy and malignant skin tissue using RGB images captured by a smartphone camera [52]. The autofluorescence maps generated for the skin malformation included in clinical testing showed good potential of using this method for pathological tissue demarcation in dermatology [52]. A previous study validated the selection of the smartphone used for this approach, and it was highlighted that the suitable devices should allow users to choose the settings for image capture [53].

The mapping of three main chromophores of skin, also employing a smartphone camera, was proved by Spigulis et al. in the study described in [54]. Illumination was ensured by a laser source which had three wavelengths corresponding to the variations in concentrations of melanin, oxyhemoglobin, and deoxyhemoglobin. The monochromatic images, generated from cumulation of RGB signals for each wavelength, were used to extract the intensity fluctuations for regions related to healthy skin and pathological tissue. Approximations were made based on Beer’s law of interactions between light and tissue, and the photon absorption path lengths in skin were estimated by Monte Carlo simulations. For nine skin malformations, including nevi, seborrheic keratosis, and hemangiomas, maps were obtained for the three chromophores (melanin, oxyhemoglobin, and deoxyhemoglobin) and the correlation with the initial diagnosis was confirmed by increased concentrations in the lesion region.

Combining autofluorescence with the chromophore mapping approach, a new prototype for skin tissue analysis was created by Lihacova et al. in [55]. The device was used in several studies to find significant differences between melanoma and non-melanoma lesions of the skin [13,55,56,57]. A parameter p’ was computed by the following formula:

$$p^{\prime }=\log\left(\frac{I_{\left(526\right)}\;\cdot \;I_{skin\left(663\right)}\;\cdot \;I_{skin\left(964\right)}}{I_{skin\left(526\right)}\;\cdot \;I_{\left(663\right)}\;\cdot \;I_{\left(964\right)}}\right),$$

where $I_{\left(526\right)},\;I_{\left(663\right)},\;I_{\left(964\right)}\;$ are the reflection images captured while the lesion is illuminated with radiation of 526, 663 and 964 nm wavelengths, respectively, and $I_{skin\left(526\right)},\;I_{skin\left(663\right)},\;\;I_{skin\left(964\right)}\;$ are the reflection images obtained with the same illumination, but of the healthy skin tissue [55]. The parameter p’ map was produced for each lesion included in the datasets, and its values were determined by the images collected while the corresponding skin area was illuminated sequentially with four wavelengths: 405 nm to induce skin autofluorescence, 526 nm to retrieve the blood absorption, 663 nm for melanin absorption evaluation, and 964 nm to gain information about the deeper layers of the skin [55,56]. The distinction between melanoma and other skin diseases was achieved by setting a threshold value for p’ and the mean of autofluorescence intensity values [55,56].

The classification of melanoma and seborrheic keratosis lesions was analyzed in the study of Bozsányi et al. [13], and a new index was introduced, demonstrating that MSI can generate significant information to complete this task. Their index was computed based on the ratio of intensity values from autofluorescence, green, and red channels of the image acquired for a skin lesion. In addition, a particle analysis was computed to identify the particle with high fluorescence values and the previous cumulative ratio was summed with the resulting product between particle number and area percentage (ratio of the area of particle with high florescence values and area of the lesion) [13]. Automated classification of different types of skin malformations was also tested using a convolutional neural network trained and validated on a reduced dataset of multispectral images [57]. The MSI device played an essential role because it made it possible to build a collection of different skin lesions: melanoma-like, pigmented benign, hyperkeratotic, and other lesions, as well as non-melanoma skin cancer such as basal cell carcinoma. As one of the first approaches for the classification of multimodal clinical images, it was concluded that further improvements to the proposed technique are still needed, especially in terms of architecture of the convolutional neural network, which should be built from the beginning rather than using the transfer learning method [57]. A Cloud-based platform has also been developed for the management of processing and storing the images acquired with this device [58]. It was proved that a secure system can be implemented to enable a fast and efficient diagnosis process [58].

Polarization is a feature of light as an electromagnetic wave and can be used to extract more information from the interaction between light and matter. Polarized light is widely used in dermatology to clarify the visualization of tissue, since it represents the source of illumination for dermoscopy. Varying the wavelengths from VIS to NIR spectrum in three different configurations of polarization, Rey-Barruso et al. [59] proposed a polarized MSI system to identify the differences between melanoma and benign nevi lesions of the skin. The collected spectral images were used to calculate first-order statistical descriptors of the angle of polarization, intensity, and orthogonal state of contrast, as parameters of polarization. They also found that melanoma is only more contrasted than benign nevi at 671 nm. Statistically significant differences were observed for short wavelengths, 414 nm, and 447 nm, and for the mean and standard deviation of the angle of polarization.

Uthoff et al. [2] demonstrated that a smartphone camera can be suitable for building a low-cost dermoscope. Their polarized multispectral imaging device has been used to acquire images of a junctional nevus and a squamous cell carcinoma skin lesion. Image processing was performed to obtain concentration maps of melanin, hemoglobin, oxyhemoglobin, and erythema. The validation is assured through the Beer-Lambert law, a theoretical model to describe the concentrations derived from the interaction between light and skin chromophores. It was seen that the edges of both analyzed skin lesions appeared modified as the wavelength of illumination increased. In addition, the clinical trial revealed that the melanin concentration was higher in the case of the junctional nevus than the squamous cell carcinoma lesion. The authors concluded that the tool they developed could significantly improve and refine the diagnosis of skin lesions. The efficiency of this prototype needs to be tested in a more rigorous way, and on a larger number of patients, but it showed that a low-cost solution for skin inspection is feasible.

Histological inspection for detecting skin cancer is currently the gold standard in dermatology field. This procedure involves ex-vivo visualization of the excised tissue after a staining process [60]. To increase the efficiency of the diagnosis method, an MSI system that uses NIR light was created by Spreinat et al. [60] and evaluated for discrimination of histological samples with three different skin diseases: melanoma, basal-cell carcinoma, and squamous-cell carcinoma. The spectral cube resulting from the acquisition process had reflectance images after tissue illumination with radiation from spectral range 900–1500 nm as components. Variance of each set of images was evaluated to examine the changes in brightness due to lesion characteristics. It was concluded that all three types of lesions can be separated from healthy tissue because substantially increased values of variance were observed for non-affected tissue despite the injured one [60].

Setiadi et al. demonstrated that skin evaluation can be performed in a fast and comprehensive way using an LED-based MSI system [61]. They performed a rigorous examination of the technical capabilities of the developed prototype. The intensity profile of each LED, the homogeneity of illumination and the linearity, resolution, and field of view of the CCD sensor were analyzed [61]. Their calibration evaluations showed that the system is suitable for dermatological clinical use, providing quality 2D maps of chromophores concentrations.

Monitoring a skin disease after treatment administration is an important task in attaining a successful healing process. A polarized MSI system was built by Van Tien et al. to inspect morphological features of psoriasis lesions, and so the wide spectrum of applications in dermatology of this imaging modality was validated [62]. Furthermore, Li et al. performed burn tissue classification using a machine learning approach on a data set of images acquired by an MSI prototype [11]. However, the results of this study were only validated on animal tissue.

For each of the prototypes described above, Table 5 summarizes the main hardware components, such as the illumination source type or the detector, as well as the acquisition time, the estimated cost, in addition to the values reported for assessment of the results of discrimination tasks in dermatology. In addition, Table 6 summarizes the main advantages and limitations of these MSI prototypes created for dermatology purposes.

For each of the prototypes described above, Table 7 summarizes the spectral range used for the illumination sources, as well as the approaches selected to capture the images of skin regions and to perform the data processing.

## 4. Discussion

The multispectral devices developed to date can be used only as an adjuvant tool in clinical investigations. They do not meet the requirements in terms of performance to be considered a standalone screening tool. The main cause of this drawback is limited specificity of the commercially available systems. A lower specificity means that a significant number of lesions are classified as being pathological than their real number. Even if higher sensitivity involves a large number of cases being correctly diagnosed, it also does not actually decrease the number of unnecessary biopsies. Raising the specificity attained should be the goal of future multispectral imaging devices. Thus, only a reduced number of false-positive results can guarantee a qualitative screening tool for dermatological purposes.

In the last decade, more prototypes of MSI systems were proposed to fulfill the known drawbacks of the initial applications and the need for a more efficient technique in skin assessment.

As a source of illumination, the produced prototypes have mostly incorporated a ring of LEDs. The ring shape is preferred because uniform illumination is required on the lesion and the sensor has to collect only the reflected light with specific wavelengths. Halogen lamps were used in only two approaches and in one study a xenon lamp was chosen. A laser module was also used in a smartphone-based system. The wavelengths of radiation used for illumination vary from visible to NIR and even extended NIR spectrum. The number of wavelengths employed in the analyzed MSI systems was between 3 and 30. Most of the devices enabled visualization of skin sample with 8 different wavelengths of light, but good results in classification of lesions process were registered even for only 3 wavelengths. The spectral narrow bands were chosen with respect to maximum and minimum absorbance for the main chromophores of the skin. Therefore, values around 525 nm were selected for blood, 660 nm for melanin, and 950 nm for deeper layers absorption evaluation. In some cases, the photobleaching phenomenon was also considered and illumination with 405 nm radiation was performed.

The range of sensors used for collecting the reflected spectra is wide, but CCD is the most frequently chosen type. The employment of smartphone cameras and the performances reached with this type of device demonstrated the high level of portability that could have been implemented.

Other hardware components crucial to the development of an MSI device are the lenses to ensure magnification and focus of the region of interest. In addition, two polarizers are needed, one for the illumination source and one for the detector to reduce the specular reflection of the superficial layer of skin. Depending on the particularities in terms of illumination and type of the sensor, additional filters or a monochromator for spectral band selection and a diffuser for lighting uniformity can be also employed. Evolved systems also integrate acquisition triggers, drivers, and controllers to ensure adjustment of radiation power, and Bluetooth connection to a server or a computer for further processing of captured images.

From the spectral images generated as output by these MSI devices, there are extracted parameters maps according to the main chromophores or a set of numeric features about the lesions. The maps serve as guidance for physicians in diagnosing procedures, while the computed values represent the characteristics used in automated classification procedures [20].

The field of view is still limited to 2 cm^2^ with the systems developed to date, but the acquisition time was reduced over time to less than 10 s. In addition, the estimated price of production of the prototypes is lower than that of available commercial systems.

Regarding the type of lesions investigated, the range was expanded to more than only melanoma and nevi lesions with basal cell carcinoma, squamous cell carcinoma, seborrheic keratosis, and hemangiomas. This fact demonstrated the possibility of refining the diagnosis in dermatology using an MSI system. The data set size is reduced for most studies conducted for each prototype, so extensive clinical validation is required.

Most of the systems presented are active MSI systems. Thus, sequential illumination is ensured by multiple sources with different wavelengths. The polarized approach has a significant popularity justified by the better performances reached by the devices which integrated it. The spectral ranges used vary considerably, even if the wavelengths corresponding to maximum or minimum absorbance of the main chromophores of skin are very often selected. In general, spectral bands from 440 to 995 nm are chosen. Only a small number of prototypes integrate the autofluorescence effect and they used wavelengths below 440. The need to explore the deepest layers of skin made the selection of exNIR spectral bands from 1000 to 1613 nm more popular.

Image processing of spectral frames captured for a skin lesion also varies across the selected studies. Using the Beer’s law approximations to extract concentrations maps of chromophores is frequent applied. The parameters used for automated classification of skin pathologies are different in each study. This means that a standard or an ideal procedure for approaching the MSI systems output has not been established.

Based on the current capabilities of MSI devices, improvements are clearly needed in terms of field of view. Capturing and analyzing large skin lesions or even multiple skin lesion in a single frame could reduce the cost and time involved in a screening medical procedure. Spectral bands from the near-infrared region should always be included as sources of light for illumination to guarantee the inspection of deeper tissues. Fusion with artificial intelligence could also facilitate and sustain a rapid and more accurate diagnosis. A large spectrum of dermatological pathologies can be easily assessed by an MSI system, but extensive clinical validation is still needed for each of them. The skin cancer detection problem is particularly important, and the specificity registered to date by MSI devices requires significant improvements. Another future perspective will be the miniaturizing of MSI systems or development as modular devices to be connected to a smartphone. This approach will favorize their integration in telemedicine applications or on self-screening to obtain a preliminary diagnosis.

## 5. Conclusions

Techniques such as OCT, CLSM, and MPT allow skin examination with micrometric resolution and reveal important cellular size features of cutaneous malformations. Except for dermoscopy, which is significantly involved in clinical procedures of dermatology field, the other methods are not frequently applied. The accuracy in dermatological diagnosis attained by all these methods is still debatable, and only dermoscopy is frequently used by all dermatologists.

Visible light, even polarized, can ensure only the visualization of superficial layers of skin, while the NIR light is more suitable for subcutaneous inspection. The sequential investigation of light from several spectral bands and skin tissue interactions is the operating principle of multispectral imaging applied in the dermatological field. Multispectral imaging devices for dermatology have shown their potential to be cost efficient and very easy to use.

In this paper, we analyzed the current capabilities of MSI devices built for skin assessment available in the market and prototypes. Both the commercial devices and the proposed prototypes described in this review demonstrated the great potential of an MSI system for improving diagnosis in dermatology. More efforts are needed to establish a hardware setting to achieve qualitative spectral images and improve spectral image processing techniques. Improvements must be focused on skin malformation assessment because the recent studies showed MSI devices as good diagnosis adjuvant tools in dermatology.

## Figures and Tables

**Table 1:** Characteristics, performance and availability of imaging techniques developed for melanoma detection.

Imaging Technique	Illumination Source, Wavelengths	Sensor	Resolution, Field of View	Depth Penetration	Accuracy, Sensitivity, Specificity	2D, 3D	Commercial Systems
Dermoscopy [5,6,17]	LED, Tungsten Halogen Lamp, 400–700 nm (polarized/unpolarized white light)	CCD, CMOS, InGaAS	10× magnification	0.1–0.3 mm	Acc: 76.1% Se: 79–93% Sp: 69–89%	2D	Dermlite (3 Gen), Delta 20 T (Heine) etc. (large variety of devices available)
CLSM [1,3,4,5,17]	Laser diode, 830 nm	CCD	T: 0.5–1 µm L: 0.2–1.5 µm A: 3–4 µm V: 1–2 µm FOV: 8 mm^2^ from 0.5 mm^2^ spliced frames.	0.2–0.3 mm	Se: 92% Sp: 70%	2D 3D	VivaScope 1500 VivaScope 3000
OCT [17,18,19]	Laser diode, 1300 nm	InGaAs	T: 3–15 µm L: 3–7.5 µm A: 3–5 µm FOV: 6 mm^2^	1.5–2 mm	Se: 74.1% Sp: 92.4%	2D 3D	VivoSight Skintell SkinDex 300
MSI [1,3,20]	LED, 400–2500 nm	CCD, CMOS, InGaAs	L: 70 µm FOV: 12–24 mm^2^	2.5 mm	Se: 78.6% Sp: 84.6%	2D	MelaFind SIAscope
MPT [5,6,17,21]	Femtosecond laser, 750–850 nm	CCD	T: 1 µm L: 0.5 µm A: 1.5 µm FOV: 0.35 × 0.35 mm^2^	0.2 mm	Se: 71–95% Sp: 69–97%	2D 3D	DermaInspect
3D Topography Fringe projection [3,22]	He-Ne Laser, 633 nm	CCD	L: 15 µm V: 2 µm 19 × 14 mm^2^	-	Se: 80% Sp: 76.7%	2D 3D	PRIMOS DermaTOP

LED—Light-Emitting Diode, CCD—Charge-Couple Device, CMOS—Complementary Metal-Oxide Semiconductor, InGaAs—Indium Gallium Arsenide cameras, Acc—Accuracy, Se—Sensitivity, Sp—Specificity, T—Total, L—Lateral, A—Axial, V—Vertical resolution, FOV—Field of view.

**Table 2:** Advantages, limitations and possible future improvements of the imaging techniques used for skin examinations.

Imaging	Advantages	Limitations	Improvements
Dermoscopy [5,6,16,17]	Standard technique used by all dermatologists. The dermoscope is easy to use and it does not extend the observation time. Accuracy of distinction between melanoma and benign lesions is improved by 50% when dermoscopy is used versus the standalone clinical evaluation. Digital dermoscopy can be performed and images of the whole body can be acquired in just 30 min. Digital images can be used to follow up the nevi over time or to observe the new skin lesion occurrences.	An accurate diagnosis is dependent on the experience of the professional. Can visualize only superficial layers of skin, up to epidermal–dermal junction. No transversal information is provided, and the depth of the excision cannot be planned. Whole-body screening involves a high quantity of data to be analyzed. Poor resolution.	Super-high magnification dermoscopy. Integrated classification using artificial intelligence. Using different or multiple sources of light. Portable/miniaturized systems to be attached to a smartphone.
CLSM [1,3,4,5,16,17]	Good correlation with transversal histological images. Assessment of pre-surgical skin tumor margins. Real time evaluation can be performed. Provide cellular details for a skin lesion. Monitoring the effectiveness of noninvasive treatments.	Limited depth penetration of skin tissue. High cost of equipment. Extensive training. False-negative readings when diagnostic features are below papillary dermis, and for inflamed lesion or nevi with high degree of dysplasia. Large amount of storage space required. Difficult to assess lesion densely pigmented.	Reducing the time of capturing the images. Building of systems that combine it with techniques such as OCT and dermoscopy. Fusion with artificial intelligence.
OCT [16,17,18,19]	Good correlation with axial histological sectioning. Refined diagnosis for BCC superficial or infiltrative. Microscopic vascular changes can be detected. High scanning speed. Refine surgical excision borders.	Limited resolution. Low specificity for early detection melanoma. Cost is high.	Improving optical resolution and contrast. Establishing diagnostic criteria. Fusion with artificial intelligence
MSI [1,3,16,20]	Good performances for benign versus malign skin lesion classification. High sensitivity. Fully automatic devices can be created. MSI devices can be used as prebiopsy tools.	Resolution is lower. Low specificity.	Miniaturizing MSI systems. Improving specificity. Extensive clinical validation.
MPT [5,6,16,17,21]	Deeper biological tissue can be viewed in a 3D image. Useful for differentiation between aging effects and pathological conditions. Subcellular resolution. Deep penetration depth.	Cell damage can occur due to three-photon excitation. High cost. Long acquisition time. Easily affected by movement artifacts.	Using in combination with other techniques, such as OCT, to improve rapid intraoperative assessment.
3D Topography Fringe projection [3,22]	Skin relief can be retrieved in 3D format. Can be used together with automated classification techniques to distinguish between benign and malignant lesions. Obtains morphological parameters of skin. Skin roughness evaluation.	Image artifacts can be generated easily by the motion of the patient during the imaging procedure.	Using in combination with other techniques, such as dermoscopy or MSI, to refine the diagnosis. Fusion with artificial intelligence.

**Table 3:** Advantages and limitations of commercial MSI systems for dermatology.

Device, Year of Release, Producer	Advantages	Limitations
MelaFind, 2010, MELA Sciences, Inc., Irvington, NY, USA [1,14,34,43,44]	Enable evaluation of level of malignity for a skin lesion. 10 spectral images used as input for a linear classification algorithm. Reduced false-negative results in discrimination between malignant and non-malignant lesions.	“Black-box” setting—The output is binary: low or high level of malignity. High false-positive results. Hight cost. Not a screening device.
MoleMate (SIAscope), 2002, Astron Clinica, Cambrige, UK [1,14,43,45]	Melanoma risk is evaluated. Mapping chromophores of skin from 8 spectral images. MoleMate algorithm is used to retrieve statistical parameters that are further use for automated classification.	“Black-box” setting—The output is binary: low or high level of melanoma. Low specificity. High cost. Not superior to dermoscopy.
DermoSight, 2017, Balter Medical AS (Balter), Bergen, Norway [36]	Discrimination between melanoma and benign tissue. Classification using a clustering method. Melanin depth estimation. Great potential for cancer detection in dermatology.	Skin cancer evaluation algorithm is not published. Further testing is still required.
Demetra, 2019, Barco, Kortrijk, Belgium [31,38]	Multispectral dermoscope. Easy to use. Facilitate mapping of the main chromophores of skin (melanin, hemoglobin). Vascular pattern is retrieved. Cloud-platform for patients’ data.	Automated classification between malignant and non-malignant lesions is under development.

**Table 4:** Features of commercial MSI systems for dermatology.

Device, Year of Release, Producer	Illumination Source, WaveLengths	Sensor	Other Hardware Components	Output	Field of view, Maximum Depth Penetration	Sensitivity, Specificity	Scan Time	Price	Data Set Range
**MelaFind**, 2010, MELA Sciences, Inc., Irvington, NY, USA [1,14,34,43,44]	LED, 430–950 nm	CMOS	Filters for 10 spectral bands, Monitor, Computer	10 multispectral images corresponding to spectral bands selected by filters	2.5 × 2.0 cm^2^, 2.5 mm	70–100%, 9.8–82.5%	<3 s	7500 $	MM, BN
**MoleMate (SIAscope),** 2002, Astron Clinica, Cambrige, UK [1,14,43,45]	LED, 400–1000 nm	CCD	Filters, Polarizers	8 multispectral images	1.2 × 2.4 cm^2^, 2 mm	81–100%, 59–91%	3 s	8000 $	MM, BN, SK, H
**DermoSight**, 2017, Balter Medical AS (Balter), Bergen, Norway [36]	LED, 365–1000 nm	3 IEEE 1394 FireWire Cameras	2 corrections Lentils, 5 mirrors, sapphire glass	30 multispectral images	2.2 cm diameter, –	97–99%, 97–93%	<10 s	–	MM, BCC, SCC, SK, BN
**Demetra**, 2019, Barco, Kortrijk, Belgium [31,38]	LED, 400–800 nm	CCD	Touchscreen Wi-Fi	3 parameter maps: Pigment map, Blood map, Scatter contrast map, a high-quality dermoscopic image	1 cm^2^, 2 mm	–	8 s	1416 $ + monthly subscription	MM, BCC, SCC, BN, D, VL

MM—melanoma, BN—benign nevi, BCC—basal cell carcinoma, SCC—squamous cell carcinoma, SK—seborrheic keratosis, H—hemangiomas, D—dermatofibroma, VL—vascular lesions.

**Table 5:** Features of multispectral imaging prototypes developed for dermatology.

Prototype Authors, Year	Illumination Source, Wavelengths	Sensor	Other Hardware Components	Output	Field of View	Se, Sp	Scan Time	Price	Dataset Range
Diebele et al., 2012 [47]	Ring of halogen lamps, 450–950 nm	Nuance Ex MSI camera	Diffuser, Polarizer film, Objective lens	Spectral images acquired for 540, 650 and 950 nm	-	94%, 89%	1–2 min	-	81 (22 MM, 59 BN)
Kapsokalyvas et al., 2013 [48]	Ring of 12 LEDs, 470, 530, 625 nm	CMOS camera	Achromatic doublet f = 30 mm, Ring shaped polarizing plate, O-ring and a glass window, Analyzer	Five images for each illumination setting and analyzer vs. polarizer positions	8 × 6.4 mm^2^	-	-	-	2 (1 BCC, 1 BN)
Pelagotti et al., 2013 [46]	Halogen lamp	CCD Camera	Interferential filters spaced at 50 nm from 350 to 1100 nm	Multispectral images	6 cm^2^	-	-	-	40 (10 MM, 2 BCC, 28 BN)
Kim et al., 2016 [50]	LED smartphone, 440–690 nm	SP camera	Plano-concave lens, Mirror, Magnifying lens, Two linear polarizers, Nine bandpass filters, motorized filters wheel	10 multispectral images	2018.96 × 4 mm^2^	-	6.5 s	<40 $	-
Delpueyo et al., 2017 [49]	Ring of 32 LEDs, 414, 447, 477, 524, 671, 735, 890, 995 nm	CCD Camera	Lens with focus at 4 cm, Two rotating polarizers	8 reflectance and 8 absorbance spectral images	15 × 20 mm^2^	91.3%, 54.5%	40 s	-	429 (290 BN, 95 MM, 44 BCC)
Spigulis et al., 2017 [54]	Flat ring laser diffuser of three pairs of compact laser modules, 448, 532, 659 nm	SP camera, (Google Nexus)	Shielding cylinder, Collector of laser beams, Ring-shaped flat diffuser, Film polarizers for camera	Three spectral images	126 mm^2^	-	-	-	9 (3 BN, 3 SK, 3 H)
Lihacova et al., 2018 [13,55,56,57]	Ring of 16 LEDs, 405, 526, 663, and 964 nm	IDS 5Mix camera	2 polarizers, 35 mm lens, 515 nm long pass filter	Parameter maps from the spectral images	20 × 20 mm^2^	75%, 100%	30 s	-	22 (12 MM, 10 DN)
Setiadi et al., 2018 [61]	Ring of 52 LEDs, 380,405, 470, 505,565, 605,660,690, 760,850, 880, 970 nm	CCD camera	C-Mount objective lens, LED driver, Controller Arduino Uno	12 multispectral images	13.3 × 10 mm^2^ or 7 × 5.25 mm^2^	-	<6 s	-	-
Van Tien et al., 2018 [62]	LEDs, 430, 530 nm White light	CMOS camera	Stabilized power source, Two polarizers, 10×—Magnification lens	3 spectral images based on scaly levels are evaluated	-	-	-	-	Psoriasis
Rey-Barroso et al., 2018 [20]	Ring of 24 LEDs, 995, 1081, 1214, 1340, 1464, 1613 nm	InGaAs camera	Short-wave infrared lens (high transmission from 800–2000 nm), Diffuser	6 spectral images in the exNIR spectrum	15 × 20 mm^2^	78.6%, 84.6%	-	-	53 (39 BN, 14 MM)
Rey-Barroso et al., 2019 [3,59]	Ring of 32 LEDs, 414, 447, 477, 524, 671, 735, 890, 995 nm	CCD camera	Lens with spectral sensitivity from 400 to 1000 nm, Two polarizers 400–700 nm (for LEDs and lens)	5 spectral images from 414–671 nm for each 0°, 45°, 90° polarization configurations	15 × 20 mm^2^	-	-	-	40 (20 BN, 20 MM)
Spreinat et al., 2020 [60]	Xenon lamp, 900–1500 nm (20 nm steps)	NIR InGaAs camera	Monochromator, Convex lens f = 150 mm, 20× Objective, Liquid light guide, Collimating adapter, Inverted microscope	2D variance images from the 3D data cube a	-	-	2.5 s	-	3 (1 MM, 1 SCC, 1 BCC)
Uthoff et al., 2020 [2]	Printed circuit board of 24 LEDs, 450, 470, 500, 530, 580, 660, 810, 940 nm	SP camera (LG G5) IR filter Removed	f = 24 mm achromatic doublet for 0.817 magnification, 3D-printed imaging guide, Orthogonal linear polarizer, Bluetooth-connected microcontroller, Android application	9 multispectral images	9.96 × 11.67 mm^2^	-	<20 s	* 40 $	-

Se—Sensitivity, Sp—Specificity, SP—smartphone, MM—melanoma, DN—dysplastic nevi, BN—benign nevi, BCC—basal cell carcinoma, SCC—squamous cell carcinoma, SK—seborrheic keratosis, H—hemangiomas, InGaAs—Indium Gallium Arsenide, * Price without the SP.

**Table 6:** Advantages and limitations of multispectral imaging prototypes developed for dermatology.

Prototype Authors, Year	Advantages	Limitations
Diebele et al., 2012 [47]	Good preliminary results for differentiation between benign versus malignant nevi. Inception for further research.	Only a limited quantity of data collected were used to build the parameter for classification. Small data set for testing.
Kapsokalyvas et al., 2013 [48]	Low-cost equipment. Introduced more information about a skin lesion than dermoscopy. Facilitate observation of pathological skin morphologies using three maps for melanin, hemoglobin, and single scattering.	Preliminary study.
Pelagotti et al., 2013 [46]	Proved that melanoma lesions are still visible at 950 nm. Great potential to estimate the depth of the lesion.	Preliminary study.
Kim et al., 2016 [50]	Modular system that can be attached to a smartphone. Android application for image acquisition and data transfer. Spectral angles classification using spectral signatures of every pixel from the region of interest of lesion. Machine learning models are used and lead to improved specificity and sensitivity. Self-monitoring approach.	No results on malignant versus benign lesions differentiation. Needs extensive clinical trials.
Delpueyo et al., 2017 [49]	Greater performances than commercially available products for malignant versus benign lesion classification. Found that textural parameters had improved detection of melanoma and basal cell carcinoma.	Lower sensitivity than MelaFind and confocal microscopy.
Spigulis et al., 2017 [54]	Proved that chromophore mapping is possible using a smartphone. Good correlation with initial diagnosis.	Very small data set used for testing. Laser source for illumination that can cause cellular damage.
Lihacova et al., 2018 [13,55,56,57]	Cromophore mapping is combined with autofluorescence for each lesion. A new parameter map is computed to discriminate between malignant and non-malignant diseases. Device used to create a collection of multimodal images of multiple skin disseas that can be input for automamted classification models.	Further research is needed for automated classification of multimodal images of skin lesions.
Setiadi et al., 2018 [61]	Proved that 2D quality maps of chromophore concentration can be created using a LED based MSI system. Suitable for dermatological examination as adjuvant tool.	Further calibration is still required.
Van Tien et al., 2018 [62]	Proved that psoriasis lesions can be monitored with a polarized MSI device.	Preliminary study. Only psoriasis lesions were evaluated.
Rey-Barroso et al., 2018 [20]	Radiation from VIS to NIR. The NIR light is used for illumination so deeper layers of skin can be accessed. Machine learning approach for classification between meanoma and benign nevi. Greater specificity than previous studies.	Only lesion smaller than 20 mm could be imaged.
Rey-Barroso et al., 2019 [3,59]	Polarized light from VIS to NIR spectrum is used to analys melanoma and benign nevi. Proved that at 671 nm melanoma lesions have more contrast than benign nevi. Statistical differences were pointed out for images captured at short wavelengths of malingnat and non-malignant lessions.	Preliminary study.
Spreinat et al., 2020 [60]	NIR light is used for histological examination of MM, BCC, and SCC lesions.	Based on changes in brightness only separation between healthy and pathological tissue can be performed. Ex-vivo procedure.

**Table 7:** The spectral ranges and strategies to assess skin lesions used by multispectral imaging prototypes.

Prototype Authors, Year	Spectral Range	Approach
Diebele et al., 2012 [47]	450–950 nm	Passive MSI system used to capture 51 images per skin lesion, but only the 540, 650, and 950 nm images were used to create a parameter to distinguish between melanoma and benign nevi lesions.
Kapsokalyvas et al., 2013 [48]	470, 530, 625 nm	Active MSI system that generates as output the melanin, blood, and contrast maps corresponding to a skin lesion.
Pelagotti et al., 2013 [46]	450–1050 nm	Passive MSI system used to create 7 spectral images per skin lesion where dermoscopic features were analyzed.
Kim et al., 2016 [50]	440–960 nm	Passive MSI modular system to be attached to a smartphone used to collect 10 spectral images of a skin lesion. The spectral signature for each lesion was compute by classification with spectral angles measure.
Delpueyo et al., 2017 [49]	414–995 nm	Active MSI system used to acquire 8 spectral images of from which the reflectance and absorbance maps were extracted for a skin lesion. 15 parameters were computed for a skin lesion analyzed and a classification algorithm was applied to discriminate between melanoma, basal cell carcinoma, and benign nevi lesions.
Spigulis et al., 2017 [54]	448, 532, 659 nm	Active MSI system with laser source of illumination used to capture 3 images for each spectral band for a skin lesion. The concentrations maps of the chromophores melanin, oxyhemoglobin, and deoxyhemoglobin were extracted using Beer’s law approach
Lihacova et al., 2018 [13,55,56,57]	405, 526, 663, 964 nm	Active MSI system used to obtain autofluorescence image (405 nm) and the other three intensity images for each wavelength of illumination for a skin lesion. Classification of lesions was performed using custom parameters for different skin pathology discrimination (melanoma versus dysplastic nevi, melanoma versus seborrheic keratosis).
Setiadi et al., 2018 [61]	380–970 nm	Active MSI system used to collect 12 spectral images per skin lesion. Analysis of quality of the collected images was performed in order to establish the efficiency of computing the chromophore mapping.
Van Tien et al., 2018 [62]	430, 530 nm, white light	Active MSI system used to obtain three images to evaluate the scaly levels of a skin region.
Rey-Barroso et al., 2018 [20]	995–1613 nm	Active MSI system used to collect 6 spectral images per skin lesion. Statistical parameters were computed for each image and their threshold values were selected for the parameters using PCA algorithm to discriminate between melanoma and benign nevi lesions. Final classification was performed using a SVM approach.
Rey-Barroso et al., 2019 [3,59]	414–995 nm	Active MSI polarized system used to acquire 5 spectral images (corresponding to 3 configurations of polarization) per skin lesion. First order statistical descriptors were computed for each polarization parameter extracted from collected images. Using the values computed, it was performed the classification between melanoma and benign nevi lesions.
Spreinat et al., 2020 [60]	900–1500 nm	Active MSI system to collect a set of images per each ex vivo histological samples of melanoma, basal cell carcinoma, and squamous-cell carcinoma lesions. Variance of each set of images was performed to examine the change in brightness between healthy and pathological tissue for each type of lesion.

## Data Availability

No new data were created or analyzed in this study. Data sharing is not applicable to this article.
